# Improving the Accuracy of Urban Environmental Quality Assessment Using Geographically-Weighted Regression Techniques

**DOI:** 10.3390/s17030528

**Published:** 2017-03-07

**Authors:** Kamil Faisal, Ahmed Shaker

**Affiliations:** 1 Department of Civil Engineering, Ryerson University, Toronto, ON M5B 2K3, Canada; ahmed.shaker@ryerson.ca; 2 Department of Geomatics, College of Environmental Design, King AbdulAziz University, P.O. Box 80200, Jeddah 21589, Saudi Arabia

**Keywords:** urban environmental quality, principal component analysis, GIS overlay, geographically-weighted regression, Pearson’s correlation coefficient

## Abstract

Urban Environmental Quality (UEQ) can be treated as a generic indicator that objectively represents the physical and socio-economic condition of the urban and built environment. The value of UEQ illustrates a sense of satisfaction to its population through assessing different environmental, urban and socio-economic parameters. This paper elucidates the use of the Geographic Information System (GIS), Principal Component Analysis (PCA) and Geographically-Weighted Regression (GWR) techniques to integrate various parameters and estimate the UEQ of two major cities in Ontario, Canada. Remote sensing, GIS and census data were first obtained to derive various environmental, urban and socio-economic parameters. The aforementioned techniques were used to integrate all of these environmental, urban and socio-economic parameters. Three key indicators, including family income, higher level of education and land value, were used as a reference to validate the outcomes derived from the integration techniques. The results were evaluated by assessing the relationship between the extracted UEQ results and the reference layers. Initial findings showed that the GWR with the spatial lag model represents an improved precision and accuracy by up to 20% with respect to those derived by using GIS overlay and PCA techniques for the City of Toronto and the City of Ottawa. The findings of the research can help the authorities and decision makers to understand the empirical relationships among environmental factors, urban morphology and real estate and decide for more environmental justice.

## 1. Introduction

The terminology “quality of life” has been continuously discussed in the literature, so as to lay a foundation to serve the subsequent quantification of Urban Environmental Quality (UEQ). Szalai [1] emphasized that quality of life represents the degree of satisfaction with life and the feeling of well-being, which can be measured by exogenous and endogenous factors. Diener and Suh [2] concluded the meaning of the quality of life by the satisfaction of life. Raphael et al. [3] further echoed and agreed that quality of life tends more to be the enjoyable degree of a person toward the important responsibilities of his/her life. However, Kamp et al. [4] described the quality of life by physical and immaterial equipment, such as health, education, justice, work, family, etc.

UEQ is the consequence of the combination of environmental parameters, including nature, open space, infrastructure, built environment, physical environment amenities and natural resources, and each parameter has its own characteristics and partial quality. Kamp et al. [4] addressed that UEQ is an essential part of the quality of life, which has basic concepts, such as health, safety and education, in addition to the physical and environmental parameters. Designing a theoretical framework of UEQ linking to the quality of life is an essential step to understand urban sustainability and human well-being. Such a framework may help to choose the parameters and the integration techniques to evaluate the multidimensional aspects of UEQ. These integration techniques are able to assess the current and predict/estimate the future UEQ, which are modelled by the municipal and city planners [4]. Thus, the assessment of UEQ can be an efficient tool to provide effective information of urban conditions, sustainable development and regional planning [5]. UEQ can be modelled using satellite remote sensing techniques through analysing multi-temporal and multi-resolution data, which are able to give a clear vision for visualizing and understanding the land cover, Land Surface Temperature (LST), water conditions and vegetation in urban areas [6,7]. Consequently, several studies in the literatures demonstrated the use of multi-source data to model and assess the UEQ [8,9,10].

Moore et al. [11] conducted a research study in three U.K. city centre areas, including Clerkenwell in London, Devonshire Quarter in Sheffield and the city centre of Manchester. The main goal of the study was to investigate and understand the UEQ in both subjective and objective bases, which mainly represent the city in mind and the city physically in reality, respectively. The case study divided the project into three sections: (1) outdoor environmental quality, which represents the physical, environmental conditions in the city; (2) perceived environmental quality, which represents the experiences of city residents; and (3) indoor environmental quality, which represents the physical and environmental conditions of residential buildings. Noise levels, carbon monoxide and air temperature were observed over a summer and winter period for the outdoor environmental quality assessment. For the perceived environmental quality, residents from each city were hired to conduct a photo survey and a semi-structured interview to assess residents’ experiences within each case study. The levels of carbon dioxide (CO${}_{2}$), carbon monoxide (CO), thermal conditions (${}^{\circ }$C) and light intensity were measured for the indoor environmental monitoring. The findings of this case study illustrated the local environmental quality maps and spatial urban environmental factors that represent the environmental quality within the city. The combination of subjective and objective approaches enabled encouraging people to think about how they understand the environment. The proposed method can provide an efficient way for residents worldwide to highlight their concerns, wishes and positive aspects of their local area to support decision makers.

Fobil et al. [12] presented a case study of UEQ in the City of Accra, Ghana. The primary goal of the study was to investigate the relationship between the urban environmental quality and death locations, which was commonly caused by malaria and infectious diarrhoea in low-income countries. First, a total of 65 environmental parameters, such as population and waste generation, water supply and sanitation, hygiene conditions and building structure material, were obtained from the Ghana Census 2000 database. The births and deaths registry in Accra provided the mortality data over the period 1998–2002. Second, Principal Component Analysis (PCA) was used to integrate the environmental parameters’ data and the mortality data of the study area. PCA was used to first compute the correlation among all pairwise parameters. Data reduction was subsequently conducted to reduce the environmental parameters. The results showed that all of the zones were labelled with good, bad or terrible environmental conditions. Third, analysis of variance was used to compare the differences in malaria and diarrhoea mortality levels in the three environmental zones. Fourth, a linear association was conducted between the environmental parameters and malaria and diarrhoea mortalities by using Generalized Linear Models (GLMs). The result demonstrated a strong relationship between environmental parameters and the mortality of malaria. However, there was no strong correlation found between environmental parameters and mortality from diarrhoea. The study illustrated that urban environmental management can be used to reduce the risk of infectious disease in low-income countries.

Lo [13] introduced the Landsat Thematic Mapper (TM) and social data to assess the quality of life in the city of Athens. The Landsat TM image was first obtained to generate the land use/land cover map and to extract biophysical information from it, including the Normalize Difference Vegetation Index (NDVI) and LST. Socio-economic data were obtained from U.S. census, including population density, per capita income, median home value and percent of college graduates. The maximum likelihood classification was implemented to extract low and high density residential areas, commercial and industrial areas, water, roads, forests and agriculture areas. Principal Component Analysis (PCA) and GIS overlay were used to integrate the land use/land cover, biophysical and socio-economic data. The results showed that NDVI has a strong correlation with per capita income, median home value and percent of college graduates. However, it indicates that NDVI has relatively low correlation with population density, land surface temperature, high density of residential areas, commercial areas and industrial areas. The study showed that the integration of land use/land cover, biophysical and social data can aid in predicting a realistic UEQ for the city.

Another representative study was found in U.S. counties, conducted by Shoff et al. [14]. The main goal of this case study was to investigate the place-specific risk factors for prenatal care utilization in the U.S. using Spatially-Lagged Geographically-Weighted Regression (GWR-SL). The dependent variable, including late or no prenatal care, was first extracted from the Women’s Health Quick Health Data Online from 1999–2001. The late or no prenatal care mainly represents the percentage of women who received prenatal care during their second or third period of pregnancy or did not receive prenatal care at all. The racial composition variables, including the percentage of black females of childbearing age, the percentage of American Indian/Alaskan Native (AIAN) females of childbearing age, the percentage of Asian females of childbearing age and the percentage of Hispanic females of childbearing age were obtained from the above mentioned health data to be included in the analysis. Additionally, the nativity status composition, including the percentage foreign-born, was obtained for the same period and included in the analysis. GWR-SL was implemented in this case study to model the spatial location of prenatal care utilization in U.S. counties. The results of the GWR-SL approach were compared with some of the existing methods, including ordinary least squares and the spatial lag regression model, and the GWR-SL approach showed a better understanding of prenatal care utilization in U.S. counties than the previously mentioned existing approaches. That is mainly because the GWR-SL approach takes into consideration the spatial nature of the data. The findings of this case study help to better estimate and understand the spatial prenatal care utilization in the U.S.

Despite the above successful attempts, the majority of the scholars mainly utilized PCA, GIS analysis or MCEtechniques to integrate UEQ parameters [12,13,15,16]. The PCA analytical technique has several potential disadvantages: (1) it produces unweighted components, which may not highlight those important parameters; (2) PCA does not work properly in nonlinear relationships; and finally, (3) the minimum number of components is indeterminable [5]. The GIS overlay method does not consider correlation among parameters, nor give weight to the parameters. MCE is a weighting process that allows decision makers to modify attribute values of the variables, which may lead to biased opinions. Numerous researchers [15,16,17,18] attempted to validate the UEQ results using e-mail questionnaire, field-based questionnaire, interviews with experts and factor analysis. However, these methods can be inaccurate to test the outcomes of UEQ; as a result, it may cause tendentious results. In this research, we attempt to fill several gaps in UEQ research by: (1) utilizing a new method to normalize the UEQ parameters; (2) introducing a new method to weight urban, environmental and socio-economic variables obtained from diversity data; and (3) proposing a new method to validate urban and environmental variables with socio-economic variables for UEQ assessment in two cities in Ontario, Canada.

## 2. Datasets

In this research, the City of Toronto and the City of Ottawa were intentionally selected as case studies due to the data availability and the rapid population growth in these two cities. The datasets used in this study include three broad categories: (1) Landsat TM satellite images; (2) GIS data layers; and (3) socio-economic data. All of the data were collected between the years 2010 and 2011 since GIS data and socio-economic are not consistently available to support the two case studies. A Landsat TM image was downloaded from the USGS Earth Explorer [19]. The spatial resolution of the Landsat images is 30 m for the multi-spectral bands and 120 m for the thermal band. However, the thermal bands were resampled to a 30-m pixel size from the source of data predominantly to align the thermal band with the multi-spectral bands [20]. The image was acquired during the summer season (July) to avoid the appearance of clouds and snow cover. On the other hand, a total of 14 GIS data layers were acquired from the Scholars GeoPortal [21] for both cities during the same duration of time. The GIS layer data include land use, population density, building density, vegetation and parks, public transportation, historical areas, Central Business District (CBD), sports area, religious and cultural zone, shopping centres, education institution, entertainment zones, crime rate and health condition. These layers were imported into the ArcGIS platform (ArcGIS, Esri, Redlands, CA, USA) for further analysis. Similar to the remote sensing data, all of the data were projected to the UTM 17 N coordinate system for the City of Toronto and the UTM 18 N coordinate system for the City of Ottawa. Lastly, the socio-economic parameters were derived based on the used census data that were obtained from the census bureau. The census bureau archives hundreds of parameters/information related to socio-economic conditions. In this research, socio-economic parameters, including education (university certificate, diploma or degree), family income and land values, were also obtained for the result validation. Table 1 summarizes the data sources being used in this study.

## 3. Methodology

**Figure 1:**
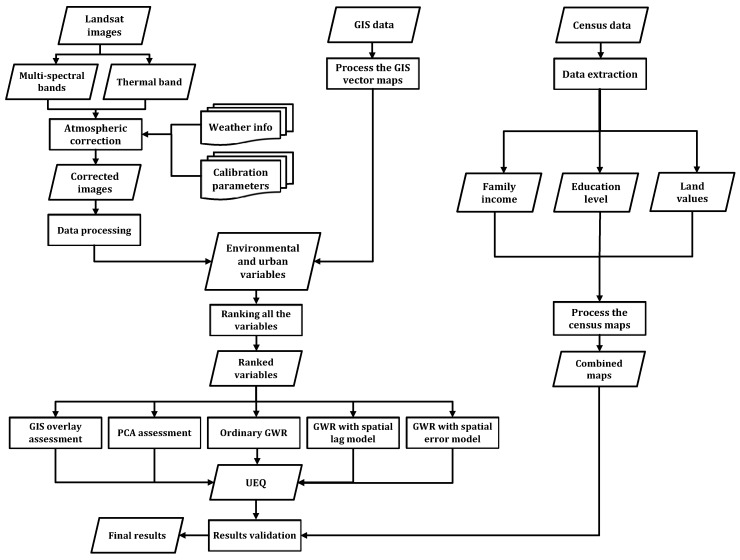
The overall workflow.

Figure 1 shows the overall workflow for the two case studies (the City of Toronto and the City of Ottawa), which can be summarized by the following steps. The Landsat images were imported into PCI Geomatics V10.1(*Geomatica*, version 10.1, PCI Geomatics, Markham, ON, Canada, 2007), clipped and then projected into the UTM coordinate system. The absolute atmospheric correction model, ATCOR2 (Atmospheric Correction and Haze Reduction), built-in PCI Geomatics software was used to compute the results for several bio-physical parameters (NDVI, NDWI, built-up index and LST) [22]. ATCOR2 was utilized to first perform radiometric calibration and to remove the effects that change the spectral characteristics of the land features [23]. Sensor parameters, including sensor type, acquisition date, Sun elevation, Sun zenith and pixel size, were obtained in addition to weather conditions (air temperature and visibility) to conduct the subsequent atmospheric correction. The calibration parameters for Landsat TM sensor (biases and gains) were also incorporated into the atmospheric correction, as is described in [24]. In this research, biophysical parameters, including NDVI, NDWI, built-up index and LST, were derived from the Landsat images. Urban, environmental and socio-economic parameters were all derived from GIS and census data to combine all of the parameters together for further analysis. The methodological contribution of this research work is to implement the GIS overlay, PCA and GWR (ordinary GWR, GWR with spatial error model and the GWR with spatial lag model) to integrate all urban, environmental and socio-economic parameters. Then, socio-economic parameters, including family income, higher education level and land values, were investigated to validate the final outcomes from the integration methods. The evaluation of the binary classifiers algorithm was performed to assess the precision and accuracy of each integration method. Based on the precision and accuracy of the integration methods, the optimal integrated method can be determined to estimate the best UEQ location in the two case studies.

### 3.1. Ranking the Parameters

**Figure 2:**
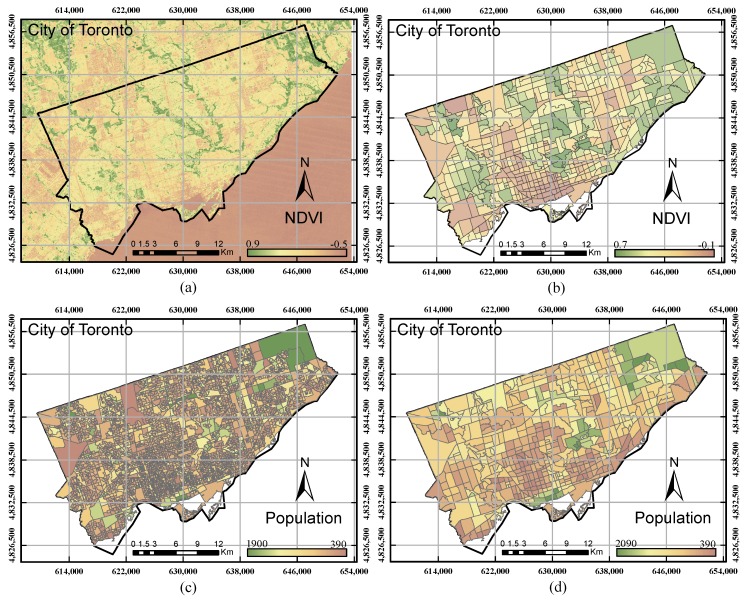
(**a**) NDVI image derived from the Landsat image (raster data); (**b**) NDVI map after transformation (vector data); (**c**) population layer at the census tract level; (**d**) population layer after transformation to the sub-neighbour level.

Since the parameters as mentioned earlier were extracted from different data sources, they may have different scale levels and cannot be combined into a particular unit. Therefore, all of the obtained data (parameters), including raster, census and GIS data, were first transformed into one scale (sub-neighbour), as shown in Figure 2. To standardize the parameters and represent the significant level of each polygon in the parameter, the Z-score method was performed for all of the parameters. The Z-score model is a statistical measurement that is able to standardize a wide range of data to represent the significant changes across data [25].

**Figure 3:**
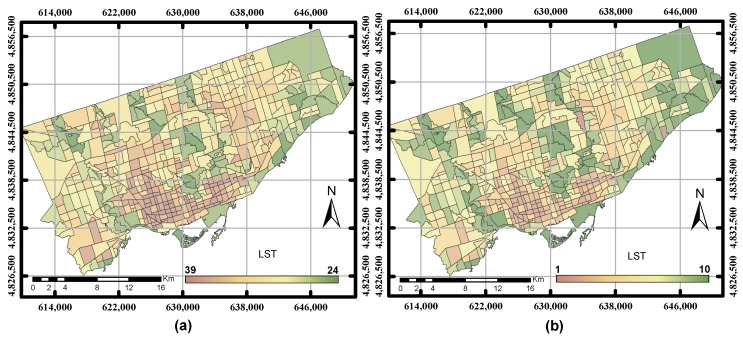
(**a**) The LST layer in degrees Celsius before ranking the parameter; (**b**) the ranking LST after the normalization.

The following Equation (1) shows the first step to normalize the parameters using the Z-score:

(1) $$Z_{i}=\left[\frac{x_{i}-\mu }{\sigma }\right]$$

where *x* is the observation values (polygons), *i* is the parameter, *μ* is the mean value of the parameter and *σ* is the standard derivation of the parameter. The second step is to use linear interpolation to rank the parameters from 1–10. The polygon within the parameter that has a high Z-score number will represent high values, for example 10. The polygon that has a low Z-score will result in a value of 1. However, for those parameters having negative relationships with respect to UEQ, such as crime rate, industrial areas, LST, etc., these parameters are inversely presented (e.g., the highest LST will take a value of 1, and the lowest LST value will get 10), as shown in Figure 3. The following Equation (2) shows how linear interpolation was calculated:

(2) $$Rank=\left[\frac{\left(Obs-Obs_{max}\right)\left(Rank_{min}-Rank_{max}\right)}{\left(Obs_{min}-Obs_{max}\right)}\right]+Rank_{max}$$

where Obs is the current observation value, $Obs_{max}$ is the maximum observation value, $Obs_{min}$ is the minimum observation value, $Rank_{max}$ is the maximum ranking value, Rank is the determined ranking value and $Rank_{min}$ is the minimum ranking value.

### 3.2. Data Integration of Multiple Environmental and Urban Parameters

Integration techniques can be used to combine remote sensing and GIS data for urban modelling and analysis [26]. Previous studies demonstrated two integration methods, mainly PCA and GIS overlay, which are able to combine various parameters from a diverse source of data. In this research work, three approaches were demonstrated to integrate the aforementioned environmental and urban parameters. These two existing approaches (PCA and GIS overlay) were first implemented, and subsequently, we investigated the use of GWR techniques (ordinary GWR, the GWR with spatial lag model and the GWR with spatial error model) to integrate all of the aforementioned parameters, which can lead to an improved estimation of UEQ.

#### 3.2.1. Geographic Information System Overlay

GIS overlay is a multi-criteria application that uses data layers for specific environmental thresholds. Remote sensing data are commonly presented as digital data in raster format. However, census data are usually stored in GIS vector format. Remote sensing data can thus be integrated with socio-economic data by converting remote sensing data from raster to vector data [27]. In this research work, the GIS overlay integration method was used to combine the urban and environmental parameters to serve for the UEQ assessment. After, we transform all of the obtained data into sub-neighbours and rank the parameters from 1–10 using Equations (1) and (2). The sum of the data layers can thus illustrate the result of UEQ.

#### 3.2.2. Principal Component Analysis

**Figure 4:**
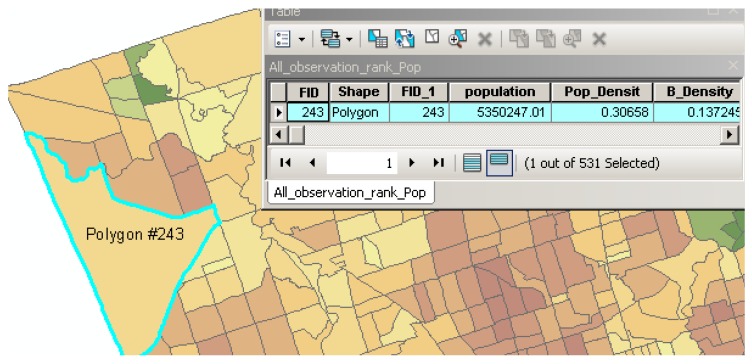
The GIS polygons of the parameters.

PCA is an analysis technique that compresses high dimensional data into a small size of data and retains most of the variance of the data [28]. PCA is commonly used in many remote sensing applications. The covariance matrix of standard PCA may not be the best option for data that have different measurement units. The correlation matrix can be used instead of the covariance matrix to standardize each parameter to the variance unit or zero means. In this research work, two case studies were conducted to assess the UEQ in the City of Toronto and the City of Ottawa, respectively. The observation values of the GIS polygons of each parameter were employed in the PCA model to determine the UEQ, as shown in Figure 4.

PCA can be computed by determining the eigenvectors and eigenvalues of the correlation matrix. The first step to compute PCA is to calculate the correlation matrix. The correlation of two random variables can be computed by using the following Equation (3):

(3) $$r_{y1,y2}=\left[\frac{cov_{\left(y_{1}i,y_{2}i\right)}}{\sigma_{\left(y_{1}i\right)}-\sigma_{\left(y_{2}i\right)}}\right]$$

where *r* is the correlation matrix for parameters $y_{1}$ and $y_{2}$, respectively, $cov_{\left(y_{1}i\right)}$ and $cov_{\left(y_{2}i\right)}$ are the covariance matrix for parameter $y_{1}$ and $y_{2}$, respectively, and $\sigma_{\left(y_{1}i\right)}$ and $\sigma_{\left(y_{2}i\right)}$ are the standard deviation for parameter $y_{1}$ and $y_{2}$, respectively, at location *i*.

The second step is to calculate the eigenvalues of the correlation matrix. The eigenvalue measures the scale of the data. The parameters that have eigenvalues greater than one will be a good rule of thumb to represent most of the variance of the data [29]. Eigenvalues can be computed by using the following Equation (4):

(4) det(A−λI)=0

where *A* is the correlation, *λ* is the eigenvalues and *I* is an *N* by *N* identity matrix.

The third step is to calculate the eigenvector of the correlation matrix. The eigenvector measures the direction of the data. Eigenvectors can be computed by using the following Equation (5):

(5) (A−λI)X=0

where *A* is the correlation matrix, *λ* is the eigenvalues and *X* is the eigenvector.

The new Obs (observation number) for the new image can be determined using the following Equation (6) [28]:

(6) $$New\left(Obs_{i}\right)=\sum_{i=1}^{n}a_{kp}*Obs_{i}$$

where $a_{kp}$ is the eigenvector for parameter *k* component *p* and Obs is the observation number in polygon *i*.

#### 3.2.3. Ordinary Geographically-Weighted Regression

One of the limitations of using PCA is that it produces unweighted components. GWR can be used to weight the spatial location of each parameter. The dependent parameter indicates the UEQ outcome, which was derived from GIS overlay method. That is mainly because the GIS overlay was found to be more emblematic for UEQ in some previous studies and one of our parallel studies [5,15]. The independent parameters are the urban and environmental parameters, which were derived from the remote sensing and GIS data, such as population density, building density, NDVI, public transportation, etc. The weight can be given to some location based on the nearness and similarity of the estimated parameters at some location. Thus, the observations that are located nearer to the estimated location would have a higher weight. However, the observations that are located far from the estimated location would have a lower weight. Assume we have a dataset that consists of a dependent variable *y* and a set of independent variables $\left(x_{1},x_{2},x_{3}\ldots x_{n}\right)$, and for each of the *i* observations in the dataset, a measurement of its position is available in a suitable coordinate system [30]. Equation (7) shows the ordinary GWR model:

(7) $$y_{i}=a_{1i}x_{1}+a_{2i}x_{2}+a_{3i}x_{3}\;\ldots .+a_{ni}x_{n}$$

where $a_{1i}\ldots a_{ni}$ refer to the coefficients that define a spatial relationship with respect to its surroundings at location *i*. The outcomes of $y_{i}$ indicates a new dependent variable if we have the dataset of the independent variables *x* at location *i*. The GWR mathematical model thus considers the weights with respect to the surroundings at location *i* to estimate coefficients $a_{1i}\ldots a_{ni}$ that define a spatial relationship with respect to its surroundings at location *i*. The following form (8) represents the coefficients $\left(a_{i}\right)$ at location *i*:

(8) $$a_{i}={\left(X^{T}W_{i}X\right)}^{-1}X^{T}W_{i}Y$$

where $W_{i}$ is a square matrix of weights relative to the position of *i* in the study area; X is the independent variables matrix; and Y is the dependent variable. The $W_{i}$ matrix captures dependency relations between the observations, which represent the geographical weights in the diagonal and 0 in its off diagonal matrix [31].

**Figure 5:**
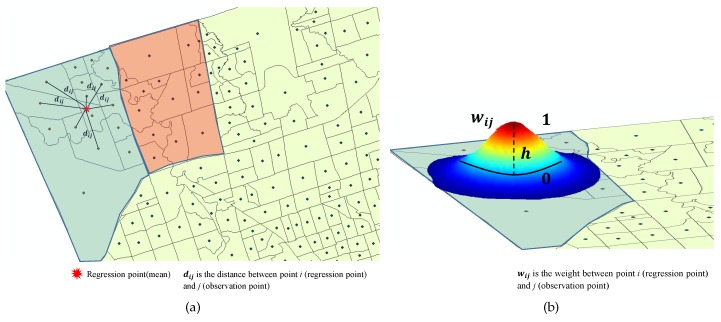
Weighted distance method. (**a**) k-nearest neighbour; (**b**) Gaussian shape kernel.

In this research work, the distance-based weights algorithm was implemented to create the diagonal weighted matrix. This method can be used to avoid non-weighted isolated polygons and polygons that are located inside other polygons. An optimum bandwidth can be defined through using certain techniques, including the Cross-Validation (CV) and Akaike Information Criterion (AIC), to derive the goodness of fit [32]. However, numerous researchers suggested different kernel functions to derive the bandwidth, such as the distance based on the taxicab geometry [33], the chamfer distance designed for a lattice or grid space [34,35], the shortest path distance [36] and the qualitative distance by translating an absolute distance metric to linguistic terms [37,38]. In this study, the first step to compute the weighted matrix is to determine the neighbours, mainly based on the k-nearest neighbour weighted method. For instance, we could generate centre points of a 10 × 10 lattice as a mean point location or regression point to measure the distances, as shown in Figure 5a. In addition, the polygons can be computed based on a weighting scheme known as a kernel, and in this study, we used the Gaussian shape kernel, as shown in Figure 5b. The following form (9) represents the weighting scheme for the distance-based method [32,39].

(9) $$w_{ij}=\exp\left[-0.5{\left(d_{ij}/h\right)}^{2}\right]$$

where $w_{ij}$ is the spatial weight between observation point *j* and regression point *i*, $d_{ij}$ is the distance between observation point *j* and regression point *i* and *h* is the kernel bandwidth defined by the distance between the regression location and the *k*-th nearest observation.

#### 3.2.4. Geographically-Weighted Regression with Spatial Lag Model

The spatial lag model is one of the dominant spatial autoregressive regression models that has been used in many research studies [40,41]. Shoff et al. [42] used the GWR with spatial lag approach to model and predict the U.S. prenatal care utilization at the county level dataset. The spatial lag model essentially heals spatial heterogeneity by including an autocorrelation coefficient and spatial weight matrix in the weighted regression model. The SLM is expressed as the following Equation (10):

(10) $$Y_{i}=\rho_{i}W_{i}y+X_{i}\beta_{i}+\epsilon$$

where Y is an *N* by 1 vector of observations on the dependent variable, *i* is the location coordinates (centroid of the county), W is an *N* by *N* specifying spatial weights matrix, which indicates the distance relationship between locations *i* and *j*, and $\rho_{i}$ is the spatial lag dependence between county level percentages of UEQ at location *i*. For a given location, say *j*, *ρ* indicates the relationship between *j*’s dependent variable (UEQ) and the dependent variable of *j*’s neighbours defined by the distance weight matrix. Positive *ρ* refers to a positive spatial autocorrelation; and if *ρ* is negative, then negative spatial autocorrelation is determined. $\beta_{i}$ is a *K* by 1 vector of regression coefficients associated with $X_{k}$ at location *i*. *ϵ* is an *N* by 1 vector of the error term.

#### 3.2.5. Geographically-Weighted Regression with Spatial Error Model

The GWR with spatial error model is appropriate when we are interested in correcting spatial autocorrelation due to the use of spatial data. In this case, the structure or spatial heterogeneity of the spatial relationship is missing. Therefore, we include the spatial autoregressive error term due to unobservable features or omitted variables that are related to locations [43]. The GWR with spatial error model is expressed as the following Equations (11) and (12):

(11) $$Y_{i}=X_{i}\beta_{i}+\epsilon$$

(12) $$\epsilon =\lambda_{i}W_{i}\epsilon +\zeta$$

where $\lambda_{i}$ is the spatial autoregressive coefficient for the error lag $W_{i}\epsilon$ and *ζ* is a vector of independent identically distributed errors.

#### 3.2.6. Accuracy Assessment

Data validation is one of the major concerns in UEQ research work. Several researchers attempted to assess the accuracy of the UEQ results using different methods, including e-mail questionnaire, field-based questionnaire, asking experts and factor analysis. Regardless of the considerable amount of e-mail surveys or field-based questionnaires, both approaches are time consuming and budget dependent. Besides, factor analysis used in the previous work was performed using the same parameters that have been incorporated to compute the UEQ, which make it unreliable and biased. Numerous UEQ studies did not perform any field survey or even results validation [11,12,13]. On the other hand, some of the literatures highlighted a high correlation between socio-economic parameters including (university certificate or diploma, family income and land values) and the quality of living [44,45,46,47]. Since there is a lack of ground reference to validate the results in this study, we propose to use these socio-economic parameters for data validation and to assess the UEQ results. All observed data of the three socio-economic parameters were normalized to be in the same scale from 1–10. Then, the sum of the socio-economic parameters can thus present the result of the reference, as shown in Table 2.

The first step to validate the results is to extract the observation’s values that are higher than the mean in each parameter and reference layer. That is mainly because in this study, we need to highlight the higher UEQ areas. Second, the evaluation of binary classifiers approach was used to evaluate the UEQ based on the following two performance measures through data interpretation: Precision and Accuracy [48].

Precision(P) is a measure that evaluates the probability that a positive outcome is correct using Equation (13):

(13) $$P=\frac{|TP|}{|TP|+|FP|}$$

Accuracy(Acc) evaluates the effectiveness of the classifier by its percentage of correct predictions using Equation (14):

(14) $$Acc=\frac{|TN|+|TP|}{|FN|+|FP|+|TN|+|TP|}$$

where TP refers to “True Positive”, which means the above mean polygons derived from the proposed method are being matched physically in the reference layer; TN refers to “True Negative”, which represents the above mean polygons that are not detected in the proposed method and the reference layer; FP refers to “False Positive”, which means the above mean polygon derived from the proposed method does not really exist in the reference layer; and FN refers to “False Negative”, which means the above mean reference polygons do not exist in the proposed method. With these three indicators, we assessed the UEQ layer from the results of each proposed method, including GIS overlay, and PCA assessed the best method for our datasets.

## 4. Results and Discussion

### 4.1. GIS Overlay Analysis

**Figure 6:**
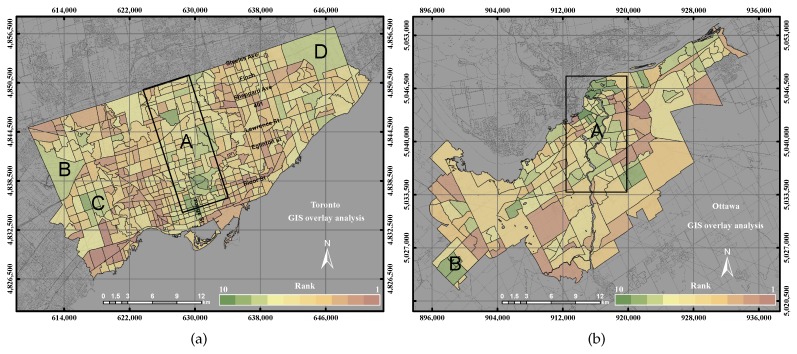
The Urban Environmental Quality (UEQ) derived using the GIS overlay method. (**a**) The UEQ in the City of Toronto; (**b**) the UEQ in the City of Ottawa.

Figure 6a shows the UEQ derived in the City of Toronto using the GIS overlay. The distribution of UEQ in the City of Toronto shows that the highest UEQ zones were found in areas (A, B, C and D) in green colour, while the lowest UEQ zones are indicated as brown colour in the city. The highest UEQ zones are the consequence of the summation of all of the positive parameters that are located within Zones A–D. However, negative values of the parameters, including crime, industrial areas and high LST, are consistently located in the brown zones within the city. In contrast, the highest values of UEQ areas were found in the high and moderate density areas, while the lowest values were found in the industrial and low density areas. Figure 6b shows the UEQ derived in the City of Ottawa using the same method. Apparently, the distribution of UEQ in the City of Ottawa showed that the highest UEQ zones were found in Zones A and B. These areas are mainly located in the down town of the city and the new urban area in Zone B. The highest values of UEQ areas were consistently found in the high and moderate density areas. However, some suburban areas located in Zone B showed high UEQ values, and that could be due to the increase of income of the household, resulting in a move to the suburbs, especially in automobile-dependent cities, such as the City of Ottawa [45,49].

### 4.2. Principal Component Analysis

Table 3 represents the correlation coefficient matrix among all of the parameters in the City of Toronto. Population density reported a moderate positive correlation coefficient with historical areas parameter (0.66), where building density showed a moderate negative correlation with green vegetation (−0.61), NDVI (−0.68), NDWI (−0.67) and a positive correlation with built-up areas (0.67) and LST (0.78). NDVI exposed a strong positive relationship with NDWI (0.88) and a moderate negative correlation with green vegetation (0.66). However, NDVI demonstrated a high negative correlation with the built-up areas parameter (−0.90) and LST (−0.80) and also revealed a moderate negative correlation with building density (−0.68). The built-up areas parameter reported a strong positive correlation with building density (0.67) and LST (0.79). The built-up areas parameter revealed a negative correlation with NDVI (−0.90) and NDWI (−0.89). NDVI stated a very high correlation with NDWI and a negative correlation with the built-up areas parameter and LST. NDVI also demonstrated a moderate negative correlation with building density, which indicates that high NDVI values represent low LST and low high building density areas with more green areas.

In the City of Ottawa, the building density parameter reported a moderate negative correlation coefficient with green vegetation (−0.61), NDVI (−0.66) and NDWI (−0.64) and a positive correlation with built-up areas (0.64) and LST (0.73). The green areas parameter also exposed a moderate negative correlation with LST. The data derived from remote sensing data, including NDVI, NDWI, the built-up areas parameter and LST, have high to moderate correlation with each other. NDVI has a high positive correlation with NDWI (0.97) and a high negative correlation with the built-up areas parameter (−0.95). However, NDVI established a moderate negative correlation with LST (−0.77). LST also showed a moderate negative correlation with the green areas parameter (−0.68) and NDWI (−0.75), but a moderate negative correlation with the built-up areas parameter (0.77). The industrial areas parameter revealed a notable moderate positive correlation with CBD (0.78) as shown in Table 4. In addition, these observations determined that the above-mentioned remote sensing parameters represented high to moderate correlation among each other. The results also indicated that there are some industrial areas located close to the down town zone that may affect the final results of the UEQ. As mentioned in Section 3.2.2, data reduction can improve the data processing and cost. Therefore, the PCA approach was used to reduce the size of the data.

**Figure 7:**
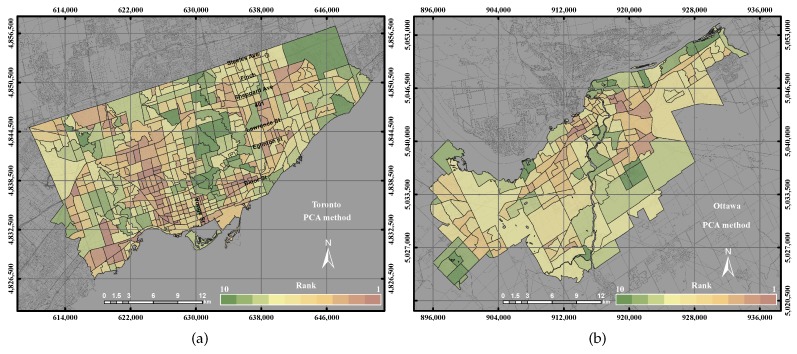
The UEQ derived using the PCA method. (**a**) The UEQ in the City of Toronto; (**b**) the UEQ in the City of Ottawa.

In this study, five components were extracted in the PCA approach for the City of Toronto, which have an eigenvalue greater than one, as shown in Figure 7. The total variance of the five components is 75% of the overall variance of the data. The preliminary analysis revealed that Component 1 has 36% of the total variance of the dataset. Component 1 shows strong positive loadings with NDVI (0.88), NDWI (0.86), building density (0.80), LST and historical areas (0.86) and strong negative loadings with LST (−0.86) and built-up areas (−0.86). In addition, Component 1 is the best to represent the green areas within the city. Component 2 reveals about 16% of the dataset, which mainly represents industrial areas with a positive correlation of 0.63 and CBD with a positive correlation of 0.76. Component 2 can be used to describe more about the urban areas. Component 3 represents 9% of the dataset, which mainly represents only sports areas with a positive correlation of (0.81). Component 4 reveals 7% of the dataset, which basically represents public transportation with a positive correlation of 0.70. The final map has a higher correlation (0.7) with the combination of Components 1 and 2. Such findings can reveal that the parameters, which are represented in Components 1 and 2, can be used to reveal the UEQ within the city.

In the City of Ottawa, six components were extracted in the PCA approach that has an eigenvalue larger than one. The outcome revealed that Components 1 and 2 have 56% and Components 3, 4, 5 and 6 have 21.1% of the total variance of the dataset. The results showed that Component 1 was highly correlated with NDVI (0.88), NDWI (0.86) and green vegetation (0.80) and has a strong negative correlation with LST (−0.84) and built-up areas (−0.86). Similar to the City of Toronto case study, Component 1 can be used predominantly to derive the green areas within the City of Ottawa. On the other hand, Component 2 detects about 18.4% of the dataset, which mainly represents industrial areas with a positive correlation of 0.72, CBD with a positive correlation of 0.66 and LST with a positive correlation of 0.70. Component 2 can be used to represent more about the urban areas. The findings of the City of Ottawa case study can reveal that the parameters, which are described in Components 1 and 2, can be used to represent the UEQ within the city.

### 4.3. Geographically-Weighted Regression

#### 4.3.1. Ordinary Geographically-Weighted Regression

**Figure 8:**
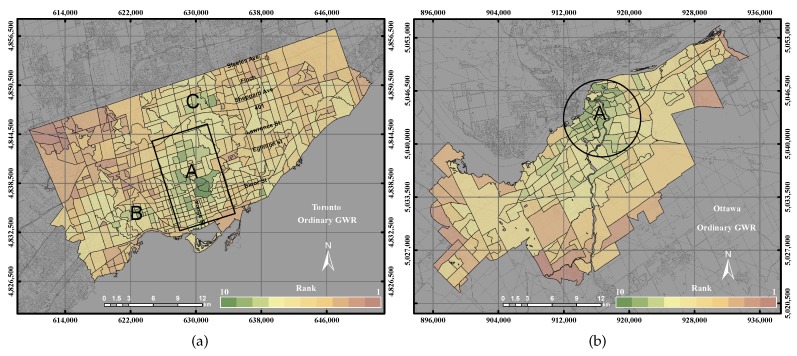
The UEQ derived using the ordinary GWR method. (**a**) The UEQ in the City of Toronto; (**b**) the UEQ in the City of Ottawa.

Figure 8a shows the derived UEQ using the ordinary GWR in the City of Toronto. The distribution of UEQ in the City of Toronto showed that the highest UEQ zones were mainly found in Zones A, B and C displayed in green colour, which are mainly located in the north and middle of town, as well as the west of the city. However, the lowest UEQ zones are located in the northwest and northeast of the city. The ordinary GWR investigates the spatial weight with respect to the city’s polygons and its surrounding polygons. Thus, the outcome showed the highest UEQ zones clustered in the middle, north and west of the city. The highest UEQ values can be ascribed by all of the positive parameters as previously mentions in the result of the GIS overlay. Figure 8b reveals the derived UEQ for the City of Ottawa using the ordinary GWR. The distribution of the higher values mainly is located in the city centre and the middle of town, as shown in Zone A in Figure 8b. The lowest UEQ zones mostly are located in the remote areas of the city. That could be again because the City of Ottawa is not a high dense city, and many positive parameters are located in the down town and middle of town of the city.

#### 4.3.2. Geographically-Weighted Regression with Spatial Lag Model

**Figure 9:**
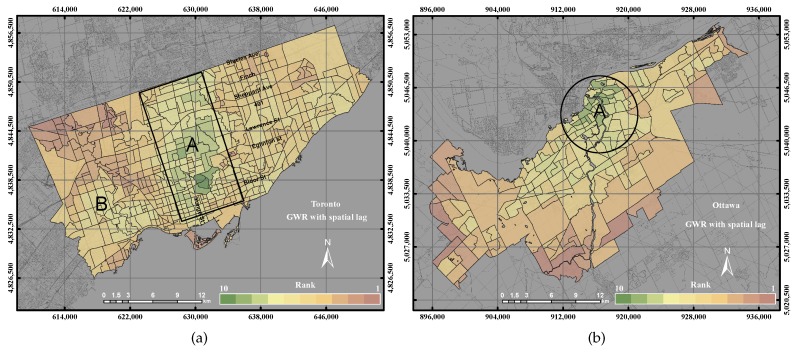
The UEQ derived using the GWR with spatial lag method. (**a**) The UEQ in the City of Toronto; (**b**) the UEQ in the City of Ottawa.

Figure 9a shows the derived UEQ using the GWR with spatial lag model in the City of Toronto. The distribution of UEQ in the City of Toronto shows that the highest UEQ zones were found in Zone A and Zone B with respect to the UEQ values within the city, while those UEQ zones with low values were located in the northwest and northeast of the city. Since the spatial lag model mainly heals the spatial heterogeneity by including an autocorrelation coefficient and spatial weights matrix in the weighted regression, thus the outcome of the spatial lag model clustered the highest UEQ zones in the middle and north of town of the city as shown in Zones A and B. That is mainly because all of the positive parameters, including (high vegetation areas, historical areas, areas supported by public transportation, low crime rate, etc.), are officially located within Zones A and B. In the City of Ottawa, the results of the GWR with spatial lag model revealed high UEQ values in the city down town and middle of town, as shown in Figure 9b. On the other hand, the lowest UEQ values are located in the suburban areas where there is a lack of public transportation, schools, hospitals and city activity.

#### 4.3.3. Geographically-Weighted Regression with Spatial Error Model

**Figure 10:**
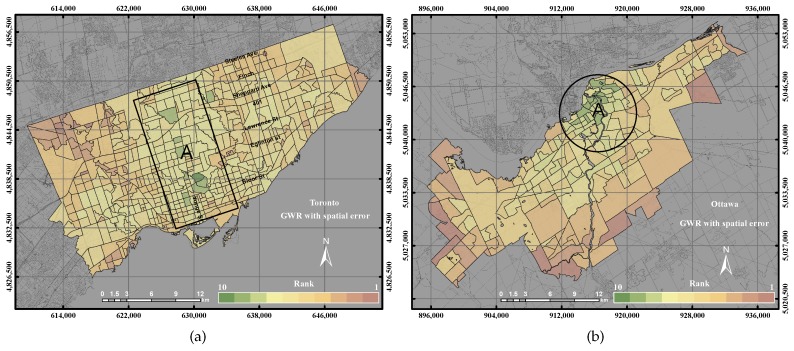
The UEQ derived using the GWR with spatial error method. (**a**) The UEQ in the City of Toronto; (**b**) the UEQ in the City of Ottawa.

The distribution of UEQ derived from using the GWR with spatial error model in the City of Toronto shows that the highest UEQ zones were clustered on Yonge Street, as shown in Figure 10a. The lowest UEQ zones are also indicated in the northwest and northeast of the city. GWR with spatial error is able to correct the spatial autocorrelation of spatial data. Thus, the outcome shows the highest UEQ zones located on the most active street within the City of Toronto. That is mainly because most of the positive parameters are located along Yonge Street. Figure 10b revealed the distribution of UEQ derived from using the GWR with the spatial error model in the City of Ottawa. The results of the GWR with spatial error showed a similar outcome as the GWR with spatial lag model, where the high UEQ values are located in the city down town and middle of town and the low UEQ values are located in the remote areas for the same reasons mentioned in the GWR with spatial lag model.

### 4.4. UEQ Results Validation

**Figure 11:**
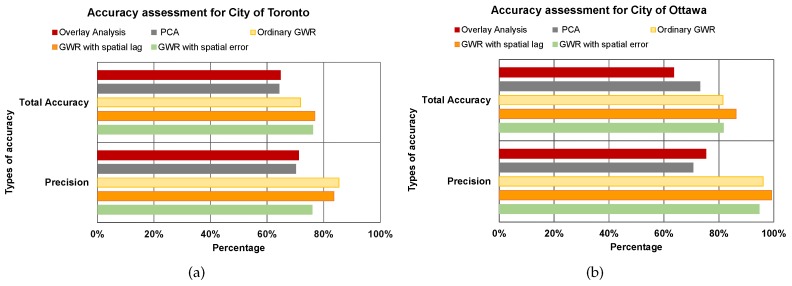
The UEQ results validation. (**a**) The City of Toronto; (**b**) the City of Ottawa.

As mentioned in Section 3.2.6, three socioeconomic parameters, including education level, family income and land values, were used to validate the UEQ results. The evaluation of binary classifiers approach was used to evaluate the UEQ. The results of GIS overlay, PCA and GWR (ordinary GWR, the GWR with spatial lag model and the GWR with spatial error model) were validated using socioeconomic parameters as a reference for this study. Since we are looking to highlight the higher UEQ areas, the mean values were used as a threshold to derive the higher UEQ areas. Figure 11 emphasizes the overall precision and accuracy of the aforementioned methods with respect to reference in this study.

**Figure 12:**
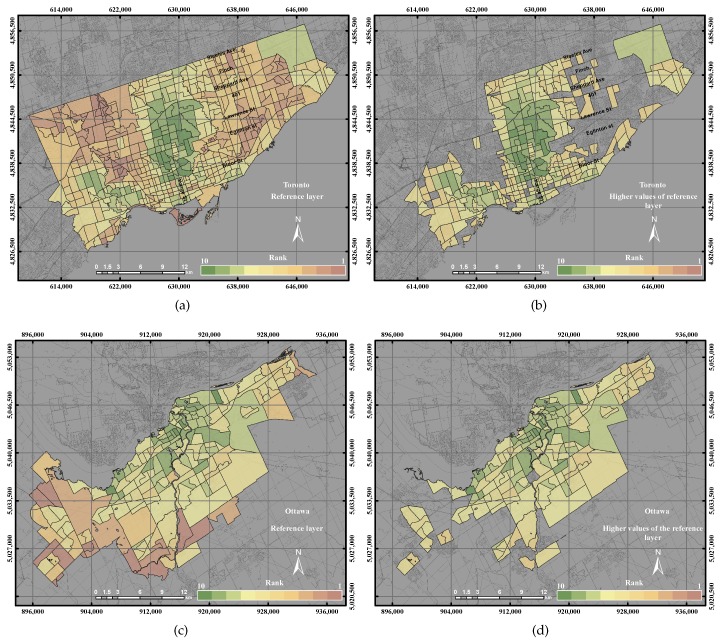
The reference layer and the higher than the mean of reference layer: (**a**) the reference layer in the City of Toronto; (**b**) the reference layer higher than the mean in the City of Toronto; (**c**) the reference layer in the City of Ottawa; (**d**) the reference layer higher than the mean in the City of Ottawa.

Figure 12 shows the reference layer and the high value of the reference layer in the two cities (the City of Toronto and the City of Ottawa). The distribution of the reference layer in the City of Toronto revealed that the highest values were found in the city centre and the west side of the city, while most of the low UEQ values were found in the northeast and northwest of the city. On the other hand, the distribution of the reference layer in the City of Ottawa revealed that the highest values were found in the city centre and the middle portions of the city, while the majority of the low UEQ values were found in the west side of the city.

**Figure 13:**
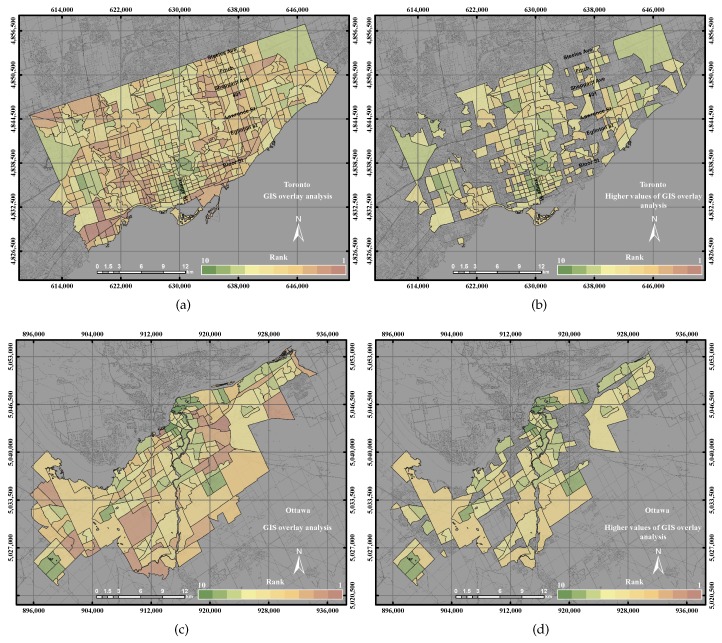
The UEQ derived using the GIS overlay method: (**a**) the derived UEQ in the City of Toronto; (**b**) UEQ zones higher than the mean in the City of Toronto; (**c**) the derived UEQ in the City of Ottawa; (**d**) UEQ zones higher than the mean in the City of Ottawa.

Figure 13 shows the GIS overlay analysis and the higher values of GIS overlay in the two cities. A few areas that have high UEQ values were located in the north and east of the city, as mentioned in Section 4.1. The precision and accuracy measured were found to be 71% and 65%, respectively, for the GIS overlay method in the City of Toronto. The precision and accuracy measured were found to be 75% and 63%, respectively, for the GIS overlay method in the City of Ottawa. That is mainly because that GIS overlay method considered all of the parameters, which have a negative and a positive relationship with respect to the reference layer. In addition, the parameters that have a negative relationship with respect to the reference layer might influence the overall result.

**Figure 14:**
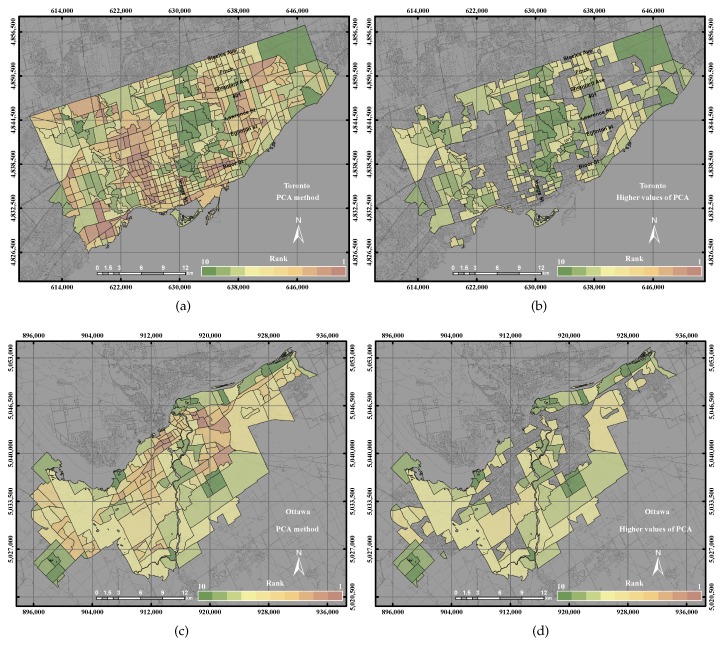
The UEQ derived using the PCA method: (**a**) the derived UEQ in the City of Toronto; (**b**) UEQ zones higher than the mean in the City of Toronto; (**c**) the derived UEQ in the City of Ottawa; (**d**) UEQ zones higher than the mean in the City of Ottawa.

Figure 14b shows higher UEQ ranking derived using the PCA in the City of Toronto and the higher values of the PCA found in the centre, north, northwest and northeast portions of the city. The overall result of the PCA method yielded a lower precision and accuracy by 1% than the GIS overlay method and 6%–15% than GWR, GWR with spatial error and GWR with spatial lag, respectively, as shown in Figure 11a. That is mainly because the PCA method does not consider 100% of the total variance. However, the rest of the methods mentioned above, including the GIS overlay method, ordinary GWR, GWR with spatial error and GWR with spatial lag, used all of the parameters. In the City of Ottawa, the PCA reported a lower precision 5% and higher accuracy by 10% than the GIS overlay method. However, PCA reported a lower precision and accuracy by 20%–25% with respect to ordinary GWR, GWR with spatial error and GWR with spatial lag for the same, as shown in Figure 11b.

**Figure 15:**
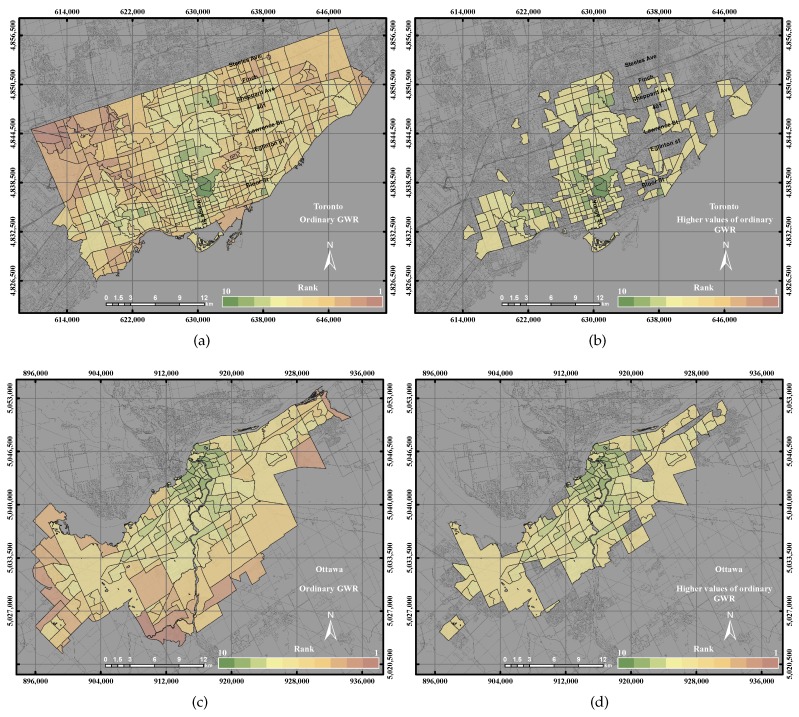
The UEQ derived using the ordinary GWR method: (**a**) the derived UEQ in the City of Toronto; (**b**) UEQ zones higher than the mean in the City of Toronto; (**c**) the derived UEQ in the City of Ottawa; (**d**) UEQ zones higher than the mean in the City of Ottawa.

In the City of Toronto, the ordinary GWR revealed higher precision and accuracy than the GIS overlay method and PCA method up to 14% and 7%, respectively, as shown in Figure 11a. Moreover, the ordinary GWR represented higher precision up to 1% than the GWR with spatial lag model and 9% precision with respect to the GWR with spatial error model. However, the accuracy of ordinary GWR reported a lower precision up to 5% with respect to the GWR with spatial lag model and the GWR with spatial error model. That occurred by investigating the ordinary GWR and the higher values of ordinary GWR with respect to the reference layer and the higher values of the reference layer. The ordinary GWR in the City of Toronto showed that the higher values of UEQ are located in the centre, north and west of the city, as shown in Figure 15b, which is visually correlated with the reference layer.

On the other hand, in the City of Ottawa, the ordinary GWR demonstrated higher precision and accuracy than the GIS overlay method and PCA method up to 20% and 17%, respectively, as shown in Figure 11b. However, the ordinary GWR revealed lower precision and accuracy up to 4% than the GWR with spatial lag model and 1% precision and accuracy with respect to the GWR with spatial error model. The ordinary GWR showed better results than GIS overlay and PCA mainly because ordinary GWR considers the spatial weight component in the method.

**Figure 16:**
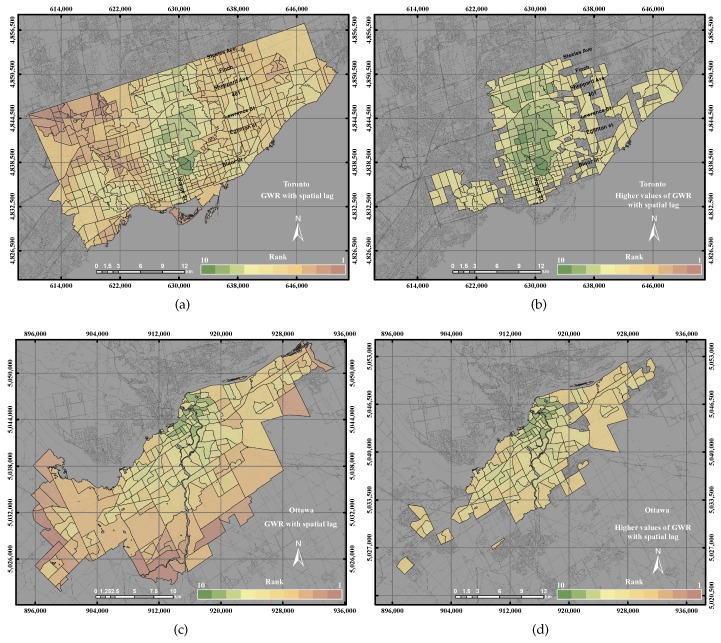
The UEQ derived using the GWR with spatial lag method: (**a**) the derived UEQ in the City of Toronto; (**b**) UEQ zones higher than the mean in the City of Toronto; (**c**) the derived UEQ in the City of Ottawa; (**d**) UEQ zones higher than the mean in the City of Ottawa.

Figure 16a,b shows the GWR with spatial lag model and the higher values of GWR with spatial lag model in the City of Toronto. As shown in Figure 16b the higher UEQ values are located in the centre, north and west of the city, which is also visually correlated with the reference layer. Thus, the precision and accuracy of the GWR with spatial lag model reported better results than GIS overlay and PCA by 15% and 8%, respectively, and 1%–5% with respect to GWR with spatial error and ordinary GWR, respectively. That is mainly because the GWR with spatial lag model adjusts the spatial heterogeneity by including an autocorrelation coefficient as mentioned previously in Section 3.2.4. The results of the City of Ottawa, on the other hand, yielded higher UEQ values that are located in the centre and the middle of the city, as the ordinary GWR. The precision and accuracy of the GWR with spatial lag model reported better results than GIS overlay and PCA by 15% and 20%, respectively, and 5% with respect to both GWR with spatial error and ordinary GWR.

**Figure 17:**
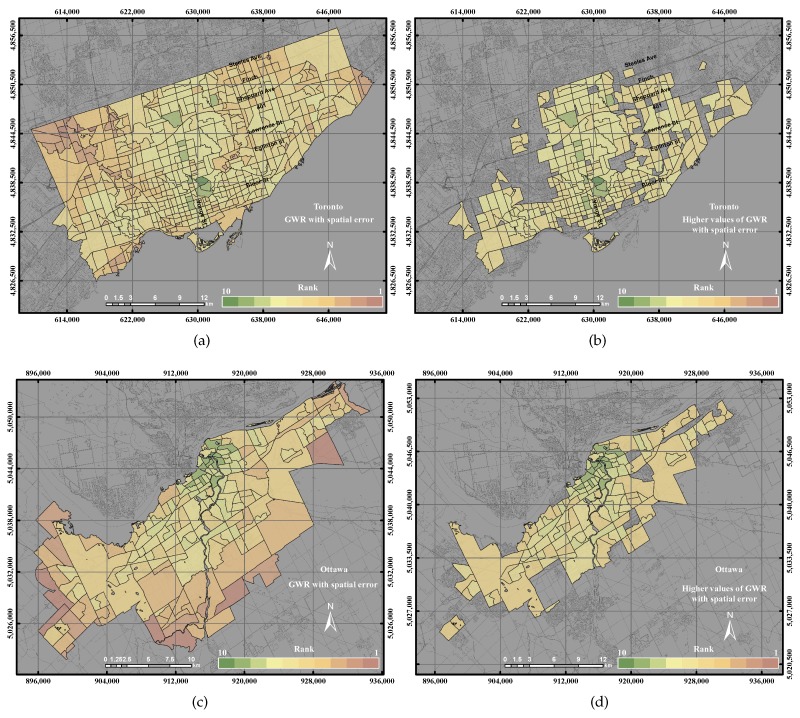
The UEQ derived using the GWR with spatial error method: (**a**) the derived UEQ in the City of Toronto; (**b**) UEQ zones higher than the mean in the City of Toronto; (**c**) the derived UEQ in the City of Ottawa; (**d**) UEQ zones higher than the mean in the City of Ottawa.

The precision and accuracy of the GWR with spatial error model both revealed 76% in the City of Toronto, but 94% and 81%, respectively, in the City of Ottawa, as shown in Figure 11. The higher values of the GWR with spatial error model in the City of Ottawa were located in the centre and the middle of the city, the same as the ordinary GWR and the GWR with spatial lag model, as shown in Figure 17d. On the other hand, the higher values of the GWR with spatial error model in the City of Toronto emerged along Yonge Street, as shown in Figure 17b. Figure 11 shows that the GWR with spatial error model revealed better precision and accuracy than GIS overlay and PCA. However, the GWR with spatial error model represents lower precision and accuracy with respect to the GWR with spatial lag model.

Besides the successful attempted methods used in this research work, there are several potential draw backs: (1) the lack of data is always an issue that may influence the final results; (2) census socioeconomic data are usually related to administrative units and can be changed in a short period, which makes it difficult to have them available worldwide; (3) remote sensing, GIS and socioeconomic data need data transformation from raster to vector or from vector to raster, which could cause an individual loss of spatial information; (4) the distance-based weighted algorithm is more applicable to a flat surface, so all of the polygons need to be projected in advance for the output to be correct; (5) the authors have previously investigated the use of linear and nonlinear regression to run the relationship between the derived UEQ with respect to the socio-economic data (reference data). However, there is no meaningful trend found in the two cities that thus reveals the inappropriate use of a linear or non-linear model in this particular case study.

## 5. Conclusions

This paper epitomizes the use of the GIS overlay, PCA and GWR techniques to assess UEQ with two case studies in Ontario, Canada. The main contribution of this research work is to investigate a new method to normalize various data derived from remote sensing, GIS and census data. New approaches of GWR techniques, including the GWR with spatial lag model and the GWR with spatial error model, were tested to assess the UEQ. The new approach was evaluated to validate the final outcomes derived from the above-mentioned methods. GWR is an intellectual framework that considers the spatial relationship among the polygons in each parameter. The GWR with spatial lag model was mainly used to provide homogeneous results by incorporating the spatial lag of the dependent variable into the GWR. Therefore, the GWR with spatial lag model is capable of producing better outcomes than other unweighted integration techniques. The GWR with spatial error model was used in this study to correct the spatial autocorrelation in the spatial data. It was found that the middle of town, north of town and southwest areas have high UEQ in the City of Toronto. However, higher UEQ was found in the city centre and middle of town within the City of Ottawa. The results illustrated that the GWR with spatial lag model significantly improved the final outcomes with respect to unweighted methods, including GIS overlay and PCA up to 15% (precision) and 8% (accuracy) in the City of Toronto and 15% (precision) and 20% (accuracy) in the City of Ottawa. Moreover, the GWR with spatial lag model also improved the final outcomes with respect to weighted methods, including ordinary GWR and GWR with spatial error model up to 1% (precision) to 5% (accuracy) in the City of Toronto and 5% (precision and accuracy) in the City of Ottawa. Thus, the GWR with spatial lag model can be used to integrate multiple parameters for UEQ purposes more accurately than the unweighted integration techniques.

Besides the success of the attempted methods used in this research work, there are several potential draw backs: (1) the lack of data is always an issue that may influence the final results; (2) census socioeconomic data are usually related to administrative units and can be changed in a short period, which makes it difficult to have them available worldwide; (3) remote sensing, GIS and socioeconomic data need data transformation from raster to vector or from vector to raster, which could cause an individual loss of spatial information; (4) the distance-based weighted algorithm is more applicable to a flat surface, so all of the polygons need to be projected in advance for the output to be correct; (5) the authors have previously investigated the use of linear and nonlinear regression to run the relationship between the derived UEQ with respect to the socio-economic data (reference data). However, there is no meaningful trend found in the two cities that thus reveals the inappropriate use of a linear or non-linear model in this particular case study.

Municipalities and decision makers can consider the proposed approach to derive the UEQ for sustainable planning in many countries. However, there is always a need for new improvement to derive better precision and accuracy in the future. Therefore, updated remote sensing and GIS data are important for better results; also, integration between weighted and GWR can be a promising method to enhance the final outcomes of UEQ; future work can be focused on modelling UEQ for an arid or cold region environment/country since there are some parameters that may not be applicable in those areas. In conclusion, remote sensing and GIS techniques are useful tools to model UEQ. Spatial weighting methods further can enhance the capability to estimate UEQ in a more accurate manner.

## Figures and Tables

**Table 1:** The data sources used in this study.

City	Landsat TM	GIS Data	Census Data
	Toronto	∘ Land Use	Socio-economic data were provided by the census bureau. Socio-economic data used in the research work include:
	Path/Row = 18/30	∘ Population Density	
	Sensor = Landsat TM	∘ Building Density	
	Date = 23 June 2011	∘ Vegetation and Parks	
		
**Toronto**	Ottawa	∘ Historical Areas	∘ Education
	Path/Row = 16/28	∘ Central Business Districts (CBD)	∘ Family Income
**Ottawa**	Sensor = Landsat TM	∘ Sports Areas	∘ Land Values
	Date = 11 September 2011	∘ Religious and Cultural Zones	
		∘ Shopping Centres	
	Remote sensing data used in this work:	∘ Education Institutions	
		∘ Entertainment Zones	
	∘ LST	∘ Crime Rate	
	∘ NDVI	∘ Health Condition	
	∘ NDWI	∘ Areas Close to Water Bodies	
	∘ NDBI and Built-up Area		

**Table 2:** The sum of the socio-economic parameters.

Polygon ID	Income	Education	Land Value	Reference Layer
1	8	5	7	20

**Table 3:** The correlation coefficient matrix among all of the parameters derived from the PCA method in the City of Toronto.

	PD	BD	PT	Veg	NDVI	NDWI	BU	LST	H	Ind	CBD	Sc	Ent	He	Rel	SP	Sea	CR	SH
PD	1.00	0.34	0.14	−0.14	−0.11	0.11	0.12	0.12	0.66	−0.04	0.08	−0.17	−0.02	0.03	−0.11	−0.04	−0.06	0.02	−0.04
BD		1.00	0.40	−0.61	−0.68	−0.67	0.67	0.78	0.44	0.07	0.39	−0.05	0.14	0.11	0.16	0.02	0.21	0.22	0.05
PT			1.00	−0.37	−0.37	−0.36	0.38	0.46	0.12	0.15	0.16	−0.09	−0.04	−0.01	0.05	−0.03	0.12	0.12	0.04
Veg				1.00	0.66	0.55	−0.56	−0.66	−0.11	−0.13	−0.09	−0.03	0.05	−0.03	−0.13	0.03	−0.30	−0.11	−0.02
NDVI					1.00	0.88	−0.90	−0.80	−0.30	−0.37	−0.37	0.02	−0.27	−0.10	−0.29	−0.09	−0.27	−0.35	−0.23
NDWI						1.00	−0.89	−0.77	−0.31	−0.39	0.37	−0.02	0.29	0.11	0.31	0.10	0.25	−0.35	0.26
BU							1.00	0.79	0.30	0.50	0.35	−0.01	0.27	0.10	0.31	0.09	0.27	0.35	0.24
LST								1.00	0.18	0.19	0.25	−0.02	0.05	0.05	0.14	0.00	0.31	0.19	0.06
H									1.00	−0.01	0.50	−0.05	0.43	0.24	0.09	0.16	−0.05	0.33	0.19
Ind										1.00	0.03	0.02	0.08	−0.01	0.31	0.05	0.06	0.12	0.14
CBD											1.00	−0.05	0.37	0.19	0.07	0.09	−0.07	0.38	0.16
Sc												1.00	0.04	0.12	0.25	0.05	0.21	0.00	0.03
Ent													1.00	0.30	0.26	0.39	0.00	0.38	0.49
He														1.00	0.30	0.49	−0.03	0.21	0.38
Rel															1.00	0.44	0.11	0.15	0.41
SP																1.00	0.02	0.18	0.62
Sea																	1.00	0.01	0.03
CR																		1.00	0.27
SH																			1.00

PD: Population Density; BD: Building Density; PT: Public Transportation; Veg: Vegetation Areas; BU: Built-up Areas; LST: LST; H: Historical Areas; Ind: Industrial Areas; Sc: School Areas; Ent: Entertainment Areas; He: Health Condition; Rel: Religion Areas; SP: Sport Areas; Sea: Areas Close to a Water Body; CR: Crime Rate Areas; SH: Shopping Areas.

**Table 4:** The correlation coefficient matrix among all of the parameters derived from the PCA method in the City of Ottawa.

	PD	BD	PT	Veg	NDVI	NDWI	BU	LST	H	Ind	CBD	Sc	Ent	He	Rel	SP	Sea	CR	SH
PD	1.00	0.46	0.29	−0.36	−0.28	−0.25	0.28	0.36	0.36	0.30	0.25	0.35	0.06	−0.02	−0.27	−0.18	−0.05	0.13	−0.11
BD		1.00	0.41	−0.61	−0.66	−0.64	0.64	0.73	0.22	0.45	0.38	0.06	0.14	0.13	0.20	−0.02	0.24	0.22	−0.05
PT			1.00	−0.31	−0.25	−0.24	−0.23	−0.37	−0.36	0.33	0.57	−0.05	0.12	0.03	0.02	0.01	0.36	−0.13	0.06
Veg				1.00	0.57	0.56	−0.57	−0.68	−0.17	−0.17	−0.10	0.04	0.04	0.01	−0.14	−0.09	−0.30	0.11	−0.01
NDVI					1.00	0.97	−0.95	−0.77	0.16	−0.40	−0.37	0.03	−0.27	−0.10	−0.30	0.01	−0.29	0.36	−0.24
NDWI						1.00	−0.96	−0.75	−0.24	−0.39	−0.34	−0.03	0.28	0.09	0.32	0.01	0.29	−0.35	0.25
BU							1.00	0.77	−0.16	0.55	0.51	0.02	−0.24	−0.09	−0.30	−0.01	−0.30	0.33	−0.23
LST								1.00	0.21	0.29	−0.23	0.02	−0.04	−0.05	−0.20	0.03	−0.35	0.23	−0.07
H									1.00	−0.29	0.43	0.02	−0.04	−0.04	−0.19	0.02	−0.34	0.21	−0.07
Ind										1.00	0.78	−0.07	0.47	0.23	0.11	0.01	−0.02	−0.33	0.17
CBD											1.00	−0.06	0.38	0.19	0.07	−0.01	−0.06	−0.36	0.15
Sc												1.00	0.04	0.10	0.23	0.14	0.21	0.02	0.04
Ent													1.00	0.27	0.19	0.18	0.01	−0.27	0.41
He														1.00	0.17	0.07	−0.03	−0.15	0.19
Rel															1.00	0.19	0.17	−0.11	0.25
SP																1.00	0.01	0.05	0.23
Sea																	1.00	−0.08	0.04
CR																		1.00	−0.12
SH																			1.00
